# Supplementary material for: A qualitative study of risks and protective factors against pregnancy among sexually-active adolescents in Soweto, South Africa
Source: PLOS Glob Public Health. 2021 Nov 10;1(11):e0000044. doi: 10.1371/journal.pgph.0000044 (PMC10021750; doi:10.1371/journal.pgph.0000044)
Supplement: S1 File — This qualitative interview guide shows the questions that guided the interviews in this study. (DOCX) [file pgph.0000044.s001.docx]

**Supplementary file 1: Interview Guide**

**Individual Interview Questions**

Hello! My name is Kate Chiseri and I am with the Birth to Twenty Cohort Research Team. I am a graduate student at Emory University in Atlanta, Georgia, United States. We are trying to gather information that will be useful in designing information and support programs for young people and we are very appreciative of your time and willingness to talk with me about the types of conversations about sexual activity you had with various people when you were an adolescent; how these helped you; what conversations you would have liked to have; with whom, and anything else you think would be useful for us to know. I would like to reiterate that this interview is confidential, and all the information that you give to us will not be shared to anyone outside of the immediate research team.

First, please tell me about yourself.

Next, I would like to ask you some questions about where you grew up.

- Who did you live with when you were a teenager? (both parents, mother, father, other family member?)
- What was the marital status of your parents when you were a teenager? (married, divorced, separated, widowed)
- Did you have siblings growing up? Did they live with you?
- Did you complete high school? (12^th^ grade) If not, what was the highest grade you completed?
- How old was your mother when she had you?

Now, please think about conversations you might have had before you had sex for the first time.

- Who did you have conversations with?
  - Who initiated the discussion? How was it approached?
  - Where did these conversations take place?
  - How old were you when these conversations started happening?
  - What did you types of things did you talk about?
  - Was protection ever spoken about? (Condoms, rubbers, female condoms, birth control)
  - How did you feel at the time? How did you react?
  - How easy was it for you to have these discussions? Were you comfortable?
  - How do you think those conversations influenced the choices you made about sex?
- Did you consider your parents knowledgeable about sex?
- IF NO discussions, why not? Do you wish you had talked about it?

Next, I am going to ask you some questions about your sexual history. Please note that when I say “sex” or “sexual activity” I am referring to vaginal or anal intercourse.

- Are your partners primarily male, female, or both?
- When you were a teenager, how often did you use protection with your partners? What types of protection did you use?
  - Never, Rarely, Sometimes, Most of the time, Always
- Have you ever been pregnant?
  - If yes, at what age did you become pregnant?
  - Have you ever had an abortion? If so, did anyone else know about the abortion?
  - How many times have you been pregnant?
- Did you ever have sex while under the influence of drugs or alcohol? If so, how often?
  - How did these substances impact the decisions you made when having sex?

Now, please think back to the time you first had sex and that experience.

- Did you and your partner talk about having sex?
- What did you talk about?
- Was protection used? If so, who initiated the use of it?
- Did you talk to anyone about your first time after it happened? If so, who?
  - What did you talk about?
- Did you consider your partner to be knowledgeable about sex?

Now, let’s talk about your relationship right now, or your most recent relationship where you were engaging in sexual activity. Which would you prefer?

- Now, as an adult, how often do you use protection with your partner(s)? What types of protection do you use?
  - Never, Rarely, Sometimes, Most of the time, Always
- How often do/did you talk about sex with your partner? (rarely, often, always, before each interaction, etc.)
- When you and your partner talk about sex, what do/did you talk about? (safe sex/condom use, feelings/emotions, what you liked, etc)
- How comfortable do you usually feel talking to your partner about sex?
- Who usually initiates/initiated discussions about sex with your partner?
- Did you ever talk about having sex without protection? Or the possibility of getting pregnant? What do you and your partner say?

As an adult, as opposed to as an adolescent, have you continued to talk with those around you about sex?

- Who do you talk to? Friends, family, partners, etc.?
- Are those conversations different as an adult as they were as an adolescent? Are you hearing similar things?
- IF NO: Why do you think that is?

(if categories of people were not addressed previously)

**Parents**

- Did your parents/guardians ever talk to you about sex or discuss anything related to sex with you?
- Would you have liked your guardians to be more open about sex? About what issues? In what ways?
- How do you think the conversations with your guardians affected your sexual decisions? (encouraged you to wait, knew how to be safe, etc.)
- Did you consider your parents to be knowledgeable about sex?

**Friends**

Did you talk to your friends about sex?

- Did you mostly talk with your male or female friends?
- What did you discuss?
- Was contraception ever spoken about? (Condoms, rubbers, female condoms, birth control)
- How did talking with your friends about sex make you feel?
- If your friends discussed their sexual experiences, how did that affect you? (Feeling uncomfortable, wanted to try it, etc.)
- Did you consider your friends to be knowledgeable about sex?

**Partner**

- Do you usually talk to your partners about sex?
- If no, why not?
- If yes, how do these conversations come up?
- What do you usually talk about?
- Are there things you would like to talk to your partner about, but don’t?

**Teachers/School Environment**

Was sex or contraception ever been spoken about in your school? (if yes)

- What information was given out at school?
- What was it like? How did you feel about it?
- Were boys and girls both taught this information?

Was sex or contraception ever been spoken about in your school? (if no)

- Would you have liked to have received information at school? Why?
- Do you think schools should teach about sex? Why or why not?

Was there a place to get rubbers at school? If so, where and how easy was it to get them?

**Community/Church/Organization members**

- Are you involved in organizations or groups, such as a church or a community center?
  - Have you had conversations with people in these groups about sex? If so, who?
    - What did you talk about?
    - Who usually initiated the conversations?
  - If not, why not?

**Your perception**

- What did you think about sex when you were an adolescent? How appropriate did you think it was for teenagers?
- GROUP 1: What are your thoughts about teenage pregnancy? How is it generally viewed in your community?
- GROUP 2: What can you tell me about teenage pregnancy in your community?

**Other sources of information**

- Did you ever visit a clinic when you were a teenager? Did you talk to anyone there about sex? Please tell me about that experience.
- Did you have conversations with family members other than parents? Please tell me about those.
- Did you go to sources other than people for information about sex when you were a teenager? (internet, TV, movies, magazines)
- Is there anyone else or any other group who you have engaged in conversations about sex with?
  - What did you talk about in these conversations?
  - Who initiated the conversations about sex?
  - Did these conversations influence your sexual behavior? If so, how?

Now, I will ask about Pregnancy and Delivery

GROUP 1: (for those who were not pregnant before age 18)

- How did conversations with anyone impact your actions and behaviors to prevent pregnancy while being sexually active?
  - With whom?
  - What did you talk about in those conversations?
  - How did these conversations influence what you did?

GROUP 2: (for those who were pregnant before age 18)

- What types of conversations occurred once you became pregnant?
  - Who did those conversations occur with?
  - What did you talk about?
  - How did these conversations differ from those you had before you became pregnant?

(Final Questions)

**(Both groups)** Who do you believe to have had the biggest influence on your sexual decision-making? Please explain why, and what they said to influence your behavior.

**(for Group 1)** What is the main reason you did not become pregnant by age 18, in an environment where some other women your age do become pregnant by age 18?

How did you achieve this?

Is there anything else you would like to say?

Do you have any questions for me?

(Thank you and closing remarks)
