# Supplementary material for: A qualitative study of risks and protective factors against pregnancy among sexually-active adolescents in Soweto, South Africa
Source: PLOS Glob Public Health. 2021 Nov 10;1(11):e0000044. doi: 10.1371/journal.pgph.0000044 (PMC10021750; doi:10.1371/journal.pgph.0000044)
Supplement: Attachment — Submitted filename: Response to Reviewers comments.docx [file pgph.0000044.s002.docx]

**Editor’s Comments**

1. In the online submission form, you indicated that "The data that support the findings of this study are available from the corresponding author on reasonable request."

All PLOS journals now require all data underlying the findings described in their manuscript to be freely available to other researchers, either 1. In a public repository, 2. Within the manuscript itself, or 3. Uploaded as supplementary information.
This policy applies to all data except where public deposition would breach compliance with the protocol approved by your research ethics board. If your data cannot be made publicly available for ethical or legal reasons (e.g., public availability would compromise patient privacy), please explain your reasons by return email and your exemption request will be escalated to the editor for approval. Your exemption request will be handled independently and will not hold up the peer review process, but will need to be resolved should your manuscript be accepted for publication. One of the Editorial team will then be in touch if there are any issues.

Authors: Thank you. We have now edited our response to Availability of data. Please see page 28, line 590, a sentence reads “All data supporting our work is provided within the manuscript.”

2. Please amend your detailed Financial Disclosure statement. This is published with the article, therefore should be completed in full sentences and contain the exact wording you wish to be published. i) State what role the funders took in the study. If the funders had no role in your study, please state: “The funders had no role in study design, data collection and analysis, decision to publish, or preparation of the manuscript.”

Authors: We have made these changes on page 28, line 592-596. A sentence reads:

**“Funding:** The study was supported by the South African Medical Research Council. DSI-NRF Centre of Excellence in Human Development at the University of the Witwatersrand, Johannesburg, South Africa and the Gates Foundation supported the Birth to Twenty Plus cohort. The funders had no role in study design, data collection and analysis, decision to publish, or preparation of the manuscript.”

Additional Editor Comments (if provided): This is a strong research article on a topic of great importance. Please consider the constructive comments below as questions as suggestions to strengthen the manuscript, at your discretion.

Authors: Thank you so much for such positive comment. We have now considered the comments and suggestions from the reviewers, and we hope the manuscript has been improved as a result.

**Authors to all reviewers:**

Thank you sincerely for taking time to review our manuscript. We appreciate the positive feedback from both reviewers. We have addressed all your comments below and we hope that we have answered all questions to your satisfaction.

**Reviewer #1:** This is such a well-written paper, engaging and scientifically sound! I wish to congratulate the authors for the effort put into producing this excellent manuscript, the methods are thoroughly described and yet concise. I noted that interviews were conducted in English- was this "piloted" first to ensure the young women completely understood the questions? I'm asking this because many times, young people would say they are comfortable with English but only to prevent being embarrassed and shamed of being unable to understand or speak English... I also noted that there was a "multi-lingual" research assistant present during the interviews- was this the note taker? it would be good to see some instances where young women expressed themselves in their own language other than English to illustrate the freedom they had to express themselves in their own language...Otherwise, this a very good and timely paper and I completely agree with authors that family institutions are and should be considered as the primary source of information for young people and thus engaging parents/caregivers in pregnancy prevention interventions is the necessary step. Congratulations again!

Authors: Thank you so much for such positive reviews and comments.

Yes, we conducted a pilot interview. However, this was conducted with staff at the research unit before the actual data collection began, and not with the adolescent girls. Please see page 6, line 144-149, a sentence reads:

“A pilot study using a focus group discussion (FGD) methodology was conducted with the Bt20+ female research staff (black Africans), at the Developmental Pathways for Health Research Unit, who understood the local languages and the context where the study was conducted. These staff were chosen based on their cultural competency and sensitivity of the topic. Staff members helped in the development of the interview guide, reviewed the questions and provided feedback on the language of interview questions and cultural appropriateness of topics. “

Given their competency and experience, engaging staff was important to help us refine our interview guide.

Indeed, we engaged a multi-lingual research assistant to ensure that participants had flexibility to speak in local languages, and hence the research assistant would help with interpretations or translations. However, all participants were able to speak in English throughout the interviews. Please see page 7, line 161-166, a sentence reads:

“Interviews were conducted in English (as all participants were able to speak in English). However, participants were allowed flexibility to express themselves in other local languages. A female, multilingual research assistant was always available during the interviews in case of need for translation and assisted in note taking. However, none of the participants used local languages during the interviews.” Thus, we do not have excerpts using local languages.

We have also clarified the role of the research assistant, see above sentence.

**Reviewer #2:** A qualitative study of risks and protective factors against pregnancy among sexually active teens in Soweto, South Africa.

Reviewer: This manuscript addresses a hugely important topic within South Africa and gives insight into the risk and protective factors of adolescent pregnancy. Overall, this manuscript is well-written and makes a considerable contribution to the field. I have a few minor suggestions below.

Introduction Line 77 – which SSA countries are you referring to? This might be important for context.

Authors: Thank you for such positive feedback.

Please see page 3, from line 63-65. We have now restructured the sentence and replaced SSA with developing countries. The sentence now reads:

“Nearly half of induced abortions in developing countries (in regions of Africa, Asia, Latin America and the Caribbean) occur among adolescent girls and young women under the age of 25 years (4).”

Reviewer: Could you add a reference for early sexual debut being defined as being before 15 years of age or add a comment/explanation in the methodology, please?

Authors: we have added a reference on the definition. See page 4, line 106 reference (22).

Reviewer: A small discussion re. some of the risks of adolescent pregnancy for adolescent mothers and their children i.e. school dropout, HIV transmission, poor birth outcomes, development outcomes for children would be useful within the introduction for contextual purposes – this could also be linked to the SDGs if useful to your overall framing.

Authors: We have considered your suggestion and added this to our introduction. See page 3, line 66-69, a sentence reads:

“Adolescent mothers are also likely to be stigmatized, drop out of school and get married or die from unsafe abortion (5,6), and could potentially have poor growth and development including that of their children – especially as their infants are more likely to be born premature and to die in the perinatal period (7).”

Also, see line 73-74, a sentence reads: “Early sexual debut is common in South Africa and has been associated with increased rate of adolescent pregnancy and risk of HIV infection (11).”

Reviewer: Methods are clear.
Given the sensitive nature of the interview guide, please could you add a note on how confidentiality was maintained during the data collection process. I appreciate the section on reflexivity – this is hugely important for contextualising results.

Authors: Thank you. We have now added a sentence on confidentiality. See page 8, line 182- 185, a sentence reads:

“Given the sensitivity of the topics that were discussed, interviews were conducted in a private room at the research unit in Soweto. Participant names were replaced with unique codes, and all information including consent forms were stored in a locked cupboard in the research unit.”

Reviewer: Results
Section 1 – sexual activity and risk: A note that this is amalgamated findings forum group 1 and group 2 would help the reader regarding the clarity of this theme.

Authors: Thank you. We have made this clarity. See page 10, line 223-224, a sentence reads:

“This section provides combined findings from group 1 and group 2 participants.”

Reviewer: Do you have data on how many children the mothers in the sample had – it might be interesting to explore whether any of these risk and protective factors related to multiple pregnancies during adolescence? Or if this was even discussed?

Authors: Thank you for this interesting question. However, this was out of scope of our study. We did not ask the number of children that the adolescent mothers in the sample had.

Reviewer: Discussion
Thought provoking and thoughtful. Implications of results for policy programming and future studies are clear. A link back to SDGs and future outcomes for adolescents may help strengthen this further but this is just a suggestion.

Authors: We agree. The first implication largely reflects on SDGs goals, and we have linked this with our recommendation. See page 25, line 535-540 reads:

“As such, policy makers should work to support adolescents through sex education and access to healthcare services and health information to help them make good choices in their lives, and this aligns with Target 3.7 of the SDGs which calls on countries “by 2030, to ensure universal access to sexual and reproductive healthcare services, including for family planning, information and education, and the integration of reproductive health into national strategies and programmes (36).”

Reviewer: An additional note in the limitations section further highlighting the impacts of having a sample which is currently 24 years of age rather than adolescents would be useful for clarity.

Authors: Thank you. See page 27, line 569-570, we have added a sentence:

“It is possible that participants might have forgotten some information that is important to understanding their experiences as adolescents.”

Reviewer: Conclusion
You refer to teenage pregnancy here and in the title. In the remainder of the manuscript, you focus on adolescent pregnancy. For clarity it may be useful to only use one term.

Authors: Thank you. We have now consistently used adolescents and not teenagers. Please see changes on the Title and in the conclusion.
